# Information Needs and Information-Seeking Behavior of Italian Neurologists: Exploratory Mixed Methods Study

**DOI:** 10.2196/14979

**Published:** 2020-04-08

**Authors:** Silvia Demergazzi, Luca Pastore, Giada Bassani, Marco Arosio, Caterina Lonati

**Affiliations:** 1 Medical Information Department Teva Italia Srl Assago Italy; 2 Research Department Doxa Pharma Srl Milan Italy; 3 Center for Preclinical Research Fondazione IRCCS Ca’ Granda Ospedale Maggiore Policlinico Milan Italy

**Keywords:** information-seeking behavior, information needs, information sources, medical information delivery, neurologists, multiple sclerosis, migraine

## Abstract

**Background:**

Current medical professions involve an extensive knowledge of the latest validated scientific data to implement disease diagnosis, therapeutic strategies, and patient care. Although clinicians can refer to a growing number and type of information sources to keep current with new scientific achievements, there are still various concerns about medical information validity, quality, and applicability into clinical practice. Novel strategies are required to identify physicians’ real-life needs with the final aim to improve modern medical information delivery.

**Objective:**

Our research used an innovative tool to collect real-time physician queries in order to investigate information needs and seeking behavior of Italian neurologists treating patients with multiple sclerosis (MS) and migraine.

**Methods:**

The study was designed as an exploratory mixed methods (ie, qualitative and quantitative) study involving 15 consecutive days of observation. A total of 50 neurologists (n=25 MS and n=25 migraine specialists) were recruited. Data were collected using an instant messaging mobile app designed for this research. At each information-seeking event, moderators triggered a computer-assisted personal interview including both semistructured interview and close-ended questions. Interactions and physician queries collected using the mobile app were coded into emerging themes by content analysis.

**Results:**

Neurologist queries were relevant to the following major themes: therapy management (36/50, 71%) and drug-related information (34/50, 67%), followed by diagnostic strategies and procedures (21/50, 42%). Quantitative analysis indicated online resources were preferentially used by clinicians (48/50, 96%) compared with offline sources (24/50, 47%). A multichannel approach, in which both online and offline sources were consulted to meet the same need, was adopted in 33% (65/198) of information-seeking events. Neurologists more likely retrieved information from online relative to offline channels (F=1.7; *P*=.01). MS specialists were 53% more likely to engage in one information-seeking event compared with migraine neurologists (risk ratio 1.54; 95% CI 1.16-2.05). MS specialists tended to be more interested in patient-related content than migraine clinicians (28% [7/25] vs 10% [2/25], *P*=.06), who conversely more likely sought information concerning therapy management (85% [21/25] vs 60% [15/25], *P*=.05). Compared with MS clinicians, migraine specialists had a harder time finding the required information, either looking at online or offline channels (F=12.5; *P*=.01) and less frequently used offline channels (30% [8/25] vs 60% [15/25] of information-seeking events, *P*=.02). When multiple sources needed to be consulted to retrieve an information item, a reduced satisfaction rate was observed both among migraine and MS specialists (single source vs multiple sources *P*=.003).

**Conclusions:**

This study provides a detailed description of real-life seeking behavior, educational needs, and information sources adopted by Italian MS and migraine neurologists. Neurologist information needs and seeking behavior reflect the specific characteristics of the specialty area in which they operate. These findings suggest identification of time- and context-specific needs of clinicians is required to design an effective medical information strategy.

## Introduction

Modern health care professionals need to consult increasing amounts of scientific content to keep current on medical science advances [1]. It has been reported that experienced physicians use as many as 2 million pieces of information to manage their patients [2]. Moreover, the recent introduction of the precision medicine (PM) model requires a deeper understanding of properties and side effects of available drugs as treatments must be tailored to the individual patient [3]. In this landscape, physicians may feel overwhelmed by the steadily expanding flow of scientific literature [4]. The massive diffusion of online scientific resources enabling health care professionals a multichannel engagement make the selection, integration, and translation of medical information into clinical practice even more complex [5]. Paradoxically, the growth of scientific evidence and access to multiple information sources do not necessarily meet clinician needs and quality standards [2,6,7].

Whereas continuing medical education is a prominent (and often mandatory) source of medical knowledge for most physicians [8], it has been shown that such programs do not often fulfill physician needs and may fail to translate into improved clinical practice patterns [9]. Indeed, the time from educational activity to real-life information needs may not allow this information to efficiently answer questions arising directly at the point of care [6,10]. In contrast, online information sources, including the open-access resource Wikipedia and social networks, have increasingly been used by physicians to quickly retrieve medical information [6,11-15]. Therefore, effective strategies for modern medical education and information delivery should be based on extensive evaluation of physicians’ real-life content needs and should likewise be prone to continuous adaptation to meet expectations in an ever-changing landscape [16].

Medical Information departments of pharmaceutical companies often deliver up-to-date, balanced, and evidence-based information on a peer-to-peer basis, answering unsolicited medical requests through different channels [16,17]. A recent survey showed that most companies in the health care sector provide some medical information [18]. Whereas reliance on industry or sponsored resources is well established in the US market, little is known on use rates for European countries and specifically for Italy.

In this study, we investigated the information needs and seeking behavior of Italian neurologists treating patients with multiple sclerosis (MS) and migraine in order to inform the content and layout of Medical Information services in our department. MS and migraine therapeutic management have evolved rather differently over the last few years. In fact, in the field of MS there were important advances, with increasing disease-modifying therapies becoming available for both progressive and relapsing-remitting MS treatment [19]. Therefore, numerous scientific educational activities and relevant online resources have been provided by pharmaceutical companies, scientific societies, and patient associations to promote MS neurologists’ continuing education [19,20]. Conversely, accurate migraine diagnosis and subclassification are still challenging due to the lack of objective gold standard diagnostic criteria [21-23]. As a consequence, neurologists treating patients with migraine must cope with the lack of robust guidelines and shortage of authoritative sources of information and educational activities [24].

Information-seeking behavior is a complex phenomenon that is contextually shaped by personal needs, learning styles, available resources, and affective components among other factors [13,25,26]. While many general theories of information seeking have been proposed, we adopted a pragmatic theoretical approach to optimize knowledge gathering for the specific purpose of developing a working app for professional content delivery to clinical physicians. For this reason we refer to the sense-making approach developed since 1972 for the study of the human use of information systems; it entails the investigation of specific situations (which define the context in which a discontinuity emerges and gives rise to information needs) and information gaps (identifying the uncertainty around a specific content) so that specific instruments can be designed for content delivery [27]. The sense-making approach proposes that the moment of communication is best described by focusing on the how the actor describes the circumstances when the information gap emerges, the content of the information gap, and its attempt to bridge this gap. Therefore, at a specific moment in time and space, an individual who self-defines as facing a gap of a particular kind may use communicating tactics of a particular kind. In a different moment facing a different gap they may use a different tactic.

This research describes the real-life seeking behavior, educational needs, and information sources of Italian MS and migraine neurologists. To the best of our knowledge, these aspects have never been explored before in this context. Our data provide an initial, exploratory step in understanding the specific information needs of neurologists and give insights on the motivation, response, and gaps in the landscape of information available for this group of users. These results could be used to design novel Medical Information strategies aimed to deliver personalized, accurate, consistent, and timely information with an omnichannel approach, allowing health care professionals to make informed decisions that can improve patient care.

## Methods

### Study Sample and Data Collection

To evaluate physician eligibility, a screener questionnaire was administered to 72 clinicians working in different health centers and hospitals throughout the Italian territory. Based on the screener results, we enrolled 50 neurologists, of whom 25 were MS specialists and 25 migraine specialists. Recruitment was planned to equally represent physicians from all Italian geographical macroregions in each specialty area.

The research was designed as an exploratory mixed methods (ie, qualitative and quantitative) study and involved 15 consecutive days of study. The observation was conducted through the Physician Line app, an instant messaging software app consisting in an instant messaging phone app based on WhatsApp that allowed physicians to share and describe the information needs experienced during their daily clinical practice and information sources used to retrieve the information needed. Participants were instructed on the Physician Line app functionalities by means of a kick-off video presentation. To improve response rate, respondents were rewarded with a cash incentive. Two expert researchers in the field of qualitative research (BG and MA) were able to initiate an interaction every time a physician sent a text message in the Physician Line app. Therefore, there was a direct interaction between physicians and moderators during the interview conducted by these means. In fact, each information-seeking event triggered a computer-assisted interview, during which a semistructured interview and two close-ended questions were administered to the physicians through the Physician Line app. The semistructured interview included 5 items capturing physician motivations and behavioral patterns (Multimedia Appendix 1). At the end of the semistructured interview, physicians were asked to rate how frequently they could retrieve appropriate content when needed and how satisfied they were about the information obtained. Ratings occurred on 5-point (from 0=not at all to 4=absolutely yes) and 4-point (from 0=not at all to 3=completely satisfied) Likert scales, respectively (Multimedia Appendix 1).

### Ethics Approval and Consent to Participate

Due to the nature of the research, no ethics committee approval was required (Italian law Decreto 8 febbraio 2013 n. 34). In fact, the study did not involve patients or lay citizens and no health intervention had been administered to participants. This study was conducted in compliance with the European Union General Data Protection Regulation 2016/679 and in line with well-established regulatory practices and procedures governing marketing research, including the Market Research Society code of conduct (2019 revision) and the Italian Code of Professional Ethics (curated by ASSIRM 2016 revision).

Physicians actively chose to participate to the study. Records collected by Doxa Pharma Srl include data retention policies, data privacy statements, permission to take part in a data collection exercise, and agreement to the processing of personal data. The interview questions were not aimed at investigating sensitive issues like religious or political beliefs or sexual orientation. Doxa Pharma Srl ensured respect of confidentiality of collected information and pseudonymization of individual answers before primary data abstraction and analysis.

### Qualitative Analysis

Physician queries and their interactions with the moderators collected with the Physician Line app were subsequently evaluated by content analysis. Following the sense-making approach, we predefined an ontology of information seeking entailing the concept of an information-seeking event described by a set of domains including situations, gaps, and tactics, namely motivation and triggers, context, information gap content, information sources, and information search strategy. An information-seeking event was defined as any action carried out by a physician in order to meet an information need (ie, consulting online sources, discussing with colleagues or sales representatives, reading a scientific article).

Each moderator abstracted relevant themes from the Physician Line app transcript with a mixed deductive-inductive content analysis of the material transcripts. Moderators precoded the transcript by highlighting codable words, sentences, or paragraphs. An initial distinction was made to discriminate motivation and triggers of information seeking and query content. The transcript was then open-coded by assigning descriptive labels to transcripts excerpts under these first two categories. Both motivation and triggers and content were further coded as follows. After consolidating redundant codes, a matrix was generated by including all codes emerging from the discussion. The codes were inductively grouped into broader categories by observing similarities of content and meaning. When disagreement occurred among coders, the item was discussed until a common taxonomy was achieved.

In a second stage, each information-seeking event was coded (for each specific motivation and content) concerning source, context, and event time (based on recording metadata). Coding of information sources was based on a predefined coding scheme (the list of codable sources is reported in Multimedia Appendix 2).

After the second stage coding, each information-seeking event was described as a vector of motivation and trigger, source, context, and event time descriptors. This information was entered in a fully codified database used for further quantitative analysis. In this context, full codification of information-seeking behavior refers to the exhaustive representation of constructs implied by the sense-making approach to describe an information-seeking event, and no additional constructs were reported.

### Statistical Analysis

We computed the absolute and relative frequency for categorical variables and means and standard deviation for continuous variables. Frequency of information-seeking events was calculated considering the total number of information-seeking events over the study period, and it was expressed by person-time incidence rate (number of information-seeking events/10 person-day). We computed confidence intervals for information-seeking event rates based on the Poisson distribution. Furthermore, we used 2-way analysis of variance to evaluate differences in information retrieval and satisfaction scores across medical specialty and information channel used. Finally, differences in proportion of content type searches across specialties were assessed by Fisher exact or chi-square tests where appropriate. Analysis was conducted with SAS 9.4 (SAS Institute Inc).

## Results

### Participants

Participants worked in different settings, encompassing small private centers and large public organizations integrating multiple operative units aimed at providing health care for a wide catchment area. Physician characteristics are reported in Table 1.

**Table 1:** Sample characteristics.

Characteristics		Migraine specialists (n=25)	Multiple sclerosis specialists (n=25)	*P* value
Age in years, mean (SD)		47.5 (7.4)	50.2 (16.1)	.44
Sex, male, n (%)		12 (48.0)	9 (34.6)	.39
**Geographical distribution, n (%)**		—	—	**.44**
	Northern regions	10 (40.0)	11 (44.0)	—
	Central regions	7 (28.0)	5 (20.0)	—
	Southern regions	8 (32.0)	9 (36.0)	—

Use of the instant messaging phone app Physician Line allowed real-time collection of relevant data without affecting the clinicians’ daily working routine; the tool was well accepted and provided physicians the opportunity to conveniently communicate with moderators.

### Qualitative Analysis

#### Content

Physician queries concerned 8 categories relevant to marketed and investigational drugs, clinical management, disease epidemiology and physiopathology, pharmaceutical companies and their activities, diagnostic procedures, patient-related topics, congress and educational opportunities, and other minor categories. Overall, 37 items could be consistently coded and classified in these 8 categories (Multimedia Appendix 3).

#### Motivations and Triggers of Information Seeking

We identified different neurologist motivations to engage in information seeking which were sorted into 2 categories. The first category included exogenous motivations (ie, triggers), external events triggering research for further information. Such events can be tentatively classified into passive and active. Specifically, passive triggers were defined as any activating content from newsletters, websites, marketing activities, or institutional or scientific communications which motivated further information seeking in the absence of a specific, preexisting information need.

I am reading a paper on NEJM about a phase 2 trial of <Drug_name> among MS patients. I got there because I just received the NEJM weekly newsletter and this topic was relevant to my practice.

N1

Conversely, active triggers were deemed to involve questions raised by patients and colleagues; emerge as noteworthy themes while the physician was actively engaged in seminars, grand round discussions, informal discussions; or were raised by clinical problems emerging in the course of a medical encounter.

One patient asked me about <Drug_name>. She has found the list of investigational drugs on Wikipedia and learned that the drug was under review by the FDA. She entered a secondary progressive course and she was worried about the clinical worsening of the disease.

N2

We also found that the need for professional growth and general scientific update rather than immediate, contingent problem-solving issues may represent a strong motivational driver for information-seeking among neurologists. In contrast to exogenous triggers, we called such experiences endogenous motivation.

Nowadays we, as physicians, must keep current on new drugs and scientific developments. Our field is growing in complexity with new insights into disease pathology and novel therapeutic options. For this reason, I browse PubMed on a weekly basis.

N3

#### Information Seeking Circumstances: Sources, Context, and Time

Physicians used both online and offline resources (a complete list is provided in Multimedia Appendix 2). Online resources were generally considered quick and easy to access, whereas offline resources were deemed to offer more chance for in-depth learning. Among online channels, the most cited search engines were Google and scientific literature repositories such as PubMed or Embase, which were preferred when looking for reliable, accurate, impartial, and complete information.

I wanted to have an overview of new drugs for MS. I did a search on PubMed by keywords and selected a few systematic reviews and editorials by reading the abstract. I will try to download the full-text in the afternoon, when I have more time.

N5

Online resources, like PubMed, were considered more convenient when accessible through mobile apps. One limitation in the use of professional scientific literature was the lack of institutional subscription to professional scientific journals since most published research requires the payment of access fees. Nonprofessional search engines such as Google were exploited for initial exploratory search or when quick answer to simple questions were needed. This was particularly true when active triggers motivated the initiation of information-seeking behavior and small pieces of information were quickly required to complement clinical decision-making or make sense of a question raised in the course of medical interactions or educational events.

During a visit I needed some info about dosing regimens for a patient with liver disease. I searched Google and easily found the leaflet of the drug online.

N6

Whereas the use of PubMed or other indexed repositories of scientific literature inherently leads to consultation of authoritative scholarly articles, the use of general public search engines such as Google requires an additional selection process on the part of physicians. Physicians reported use of institutional and noninstitutional websites, portals specializing in scientific dissemination, medical content websites, social media and blogs, and professional or patient discussion forums depending on the type and content of the piece of information searched for. Despite this extensive and multifaceted use, responders pointed out the difficulty of evaluating validity and reliability of online contents retrieved by such means. For this reason, in some cases portals of governmental institutions and scientific societies were used to find guidelines and specific authoritative grey literature material.

I received notice of definitive approval of <Drug_name>. I consulted ECTRIMS library to learn more about the drug.

N7

Offline resources included books, seminars, roundtables, workshops, educational events, and practical training. By their very nature such offline materials and events were considered authoritative sources and were used to satisfy needs related to professional development, gain an in-depth understanding of a disease, and learn new complex skills. A list of offline resources mentioned is reported in Multimedia Appendix 2.

Congresses are great opportunities to expand my network, have a grasp of current developments in the field, and improve my understanding of the disease and new drugs.

N7

### Quantitative Analysis

#### Frequency and Distribution of Information-Seeking Events

Over the 15 days of study, a total of 198 information-seeking events were collected corresponding to 2.64 information-seeking events/10 person-days (95% CI 2.29-3.28). More specifically, 120 information-seeking events (61%; 3.69 events/10 person-days; 95% CI 3.06-4.40) were sent by MS specialists, while 78 (39%; 2.4 events/10 person-days; 95% CI 1.91-2.98) were sent by migraine neurologists. Hence, MS specialists were 53% more likely to engage in one information-seeking event compared with migraine specialists (risk ratio 1.54; 95% CI 1.16-2.05). Each information-seeking event included an average of 1.98 different information searches (95% CI 1.79-2.18), for a total of 392 needs recorded.

#### Distribution of the Expressed Information Need Categories

**Figure 1:**
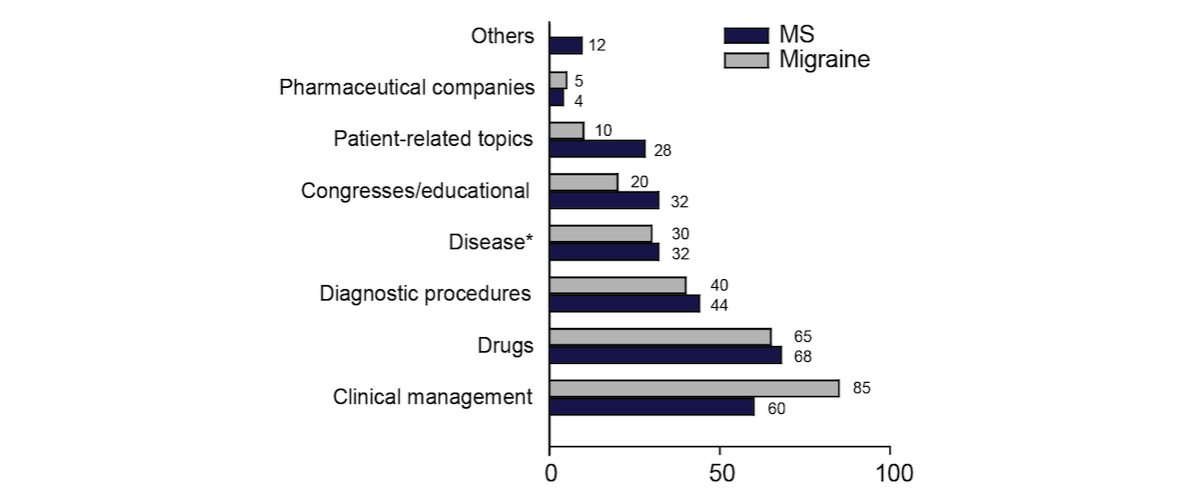
Distribution of neurologists’ major information needs. Information needs expressed by neurologists treating multiple sclerosis (n=25) and migraine (n=25) were grouped into 9 major categories by content analysis. Bars denote the share of physicians (%) reporting information seeking for each major category. *Disease epidemiology and physiopathology. MS: multiple sclerosis.

Overall, the majority of physician expressed at least one need concerning therapy management (36/50, 71%), followed by drug-related content (34/50, 67%), diagnostic strategies and procedures (21/50, 42%), disease-related content (16/50, 31%), congresses and educational opportunities (14/50, 27%), patient-related content (10/50, 20%) and other/miscellaneous including administrative issues, pharmacoeconomics (2/50, 4%), pharmaceutical companies (2/50, 4%), topical issues (1/50, 2%), and unclassified content (5/50, 10%). Distribution of major information needs was slightly different across specialties (Figure 1). Migraine specialists tended to seek information concerning therapy management more often than MS specialists (85% [21/25] vs 60% [15/25], *P*=.05); on the other hand, MS specialists tended to be more interested in patient-related content compared with migraine specialists (28% [7/25] vs 8% [2/25], *P*=.06).

#### Information Sources

**Figure 2:**
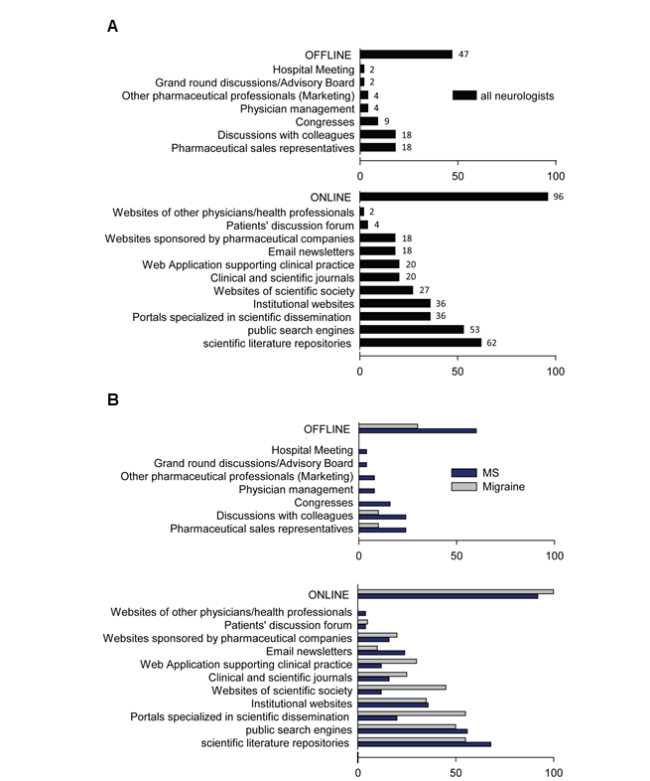
Distribution of information sources used by neurologists for information seeking. A. Channels used by neurologists (n=50) for information seeking. B. Source utilization across specialties (MS specialists, N=25, and migraine specialists, n=25). Bars denote the share of physicians (%) using each specific source for information seeking. MS: multiple sclerosis.

As shown in Figure 2A, online resources were used by the majority of clinicians (48/50, 96%), while offline resources were less often consulted (24/50, 47%). Figure 2B shows details on sources used across specialties. Migraine specialists used offline channels less frequently compared with MS neurologists (30% [8/25] vs 60% [15/25], *P*=.02).

Overall, an initial adoption of a Web search query did not exclude subsequent use of offline channels and vice versa; indeed, online and offline channels were frequently used in combination (33% [65/198] of the information seeking events), adopting a multichannel approach. Of interest, migraine specialists more likely engaged a multichannel search than MS specialists (43% [34/78] vs 27% [32/120] of the information-seeking events, *P*=.03). An explanation of this behavior can be found in the analysis of Physician Line app data, which revealed that multichannel search was adopted for complex issues needing articulated responses or when the desired information was not available in a single authoritative source. Especially in the migraine field, this implied adopting an iterative tree-like search strategy, in which answers to the original questions raised further information needs that triggered further information-seeking behaviors.

We found tentative evidence that different sources of information were selected to satisfy different information needs (Multimedia Appendix 4). For example, PubMed and other professional repositories of the scientific literature were used more often than public search engines when physicians were looking for information about disease epidemiology and physiopathology (39% [18/46] vs 11% [5x/46], *P*=.02) and diagnostic procedures (21% [7/34] vs 12% [4/34], *P*=.16). Conversely, public search engines tended to be used more frequently than professional scientific repositories to find out about congresses (9% [2/23] vs 0 [0/23], *P*=.07) and patient-related topics (31% [5/16] vs 19% [3/16], *P*=.07). Finally, professional and general search engines were equally used to look for information about drugs (18% [17/96] vs 19% [18/96]) and “clinical management” (20% [34/169] vs 19% [32/169]). Websites of scientific societies or institutions were more likely searched to retrieve information about congresses (8/23, 35%), disease epidemiology and physiopathology (6/46, 13%), and diagnostic procedures (6/34, 18%) compared with other themes (2% [3/169] for “clinical management”; 1% [1/96] for “drugs,” *P*=.01).

#### Information Retrieval Rating

**Figure 3:**
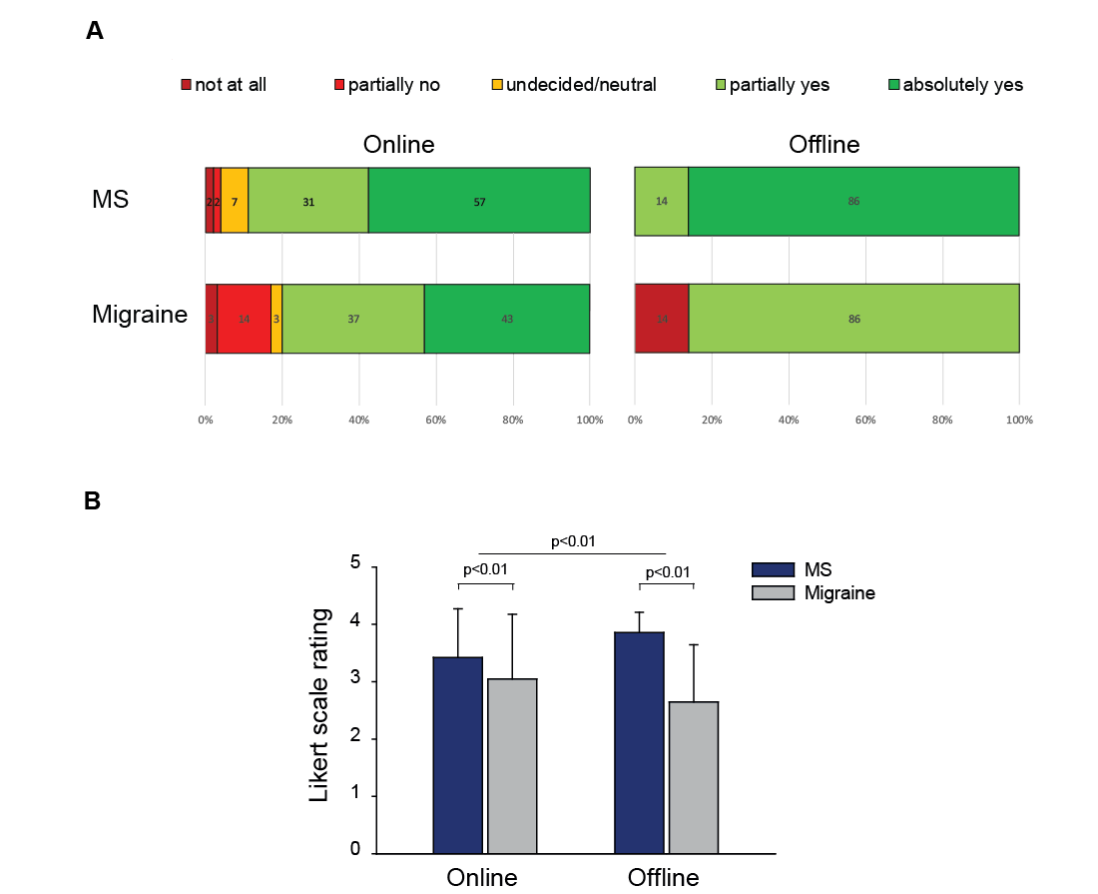
Information retrieval. A. Distribution of information retrieval ratings by information channel type and medical specialty (MS specialists, n=25, and migraine specialists, n=25). Physicians were asked to rate if they could retrieve the needed information. Diagrams show percentage of information-seeking events for each rating score of the 5-point Likert scale used (from 0=not at all to 4=absolutely yes); B. Differences in average information retrieval scores across specialty area and information channel and medical specialty. *P* values refer to 2-way analysis of variance. MS: multiple sclerosis.

Data collected with the Physician Line app indicate that the information needed was available in at least one resource for most physicians. Average information retrieval rating was 3.27 (SD 0.99) indicating that physicians most often retrieved the information they needed. In very few instances, clinicians reported they did not find the information needed (7% [26/392] of information needs, Figure 3A). Additionally, physicians more likely retrieved information from online versus offline channels (Figure 3B, *F*=1.7; *P*=.01). Migraine specialists had a harder time finding answers to their questions compared with MS neurologists, either looking at online or offline channels (Figure 3B, omnibus test, *F*=12.5; *P*=.01).

#### Satisfaction About Information Quality

**Figure 4:**
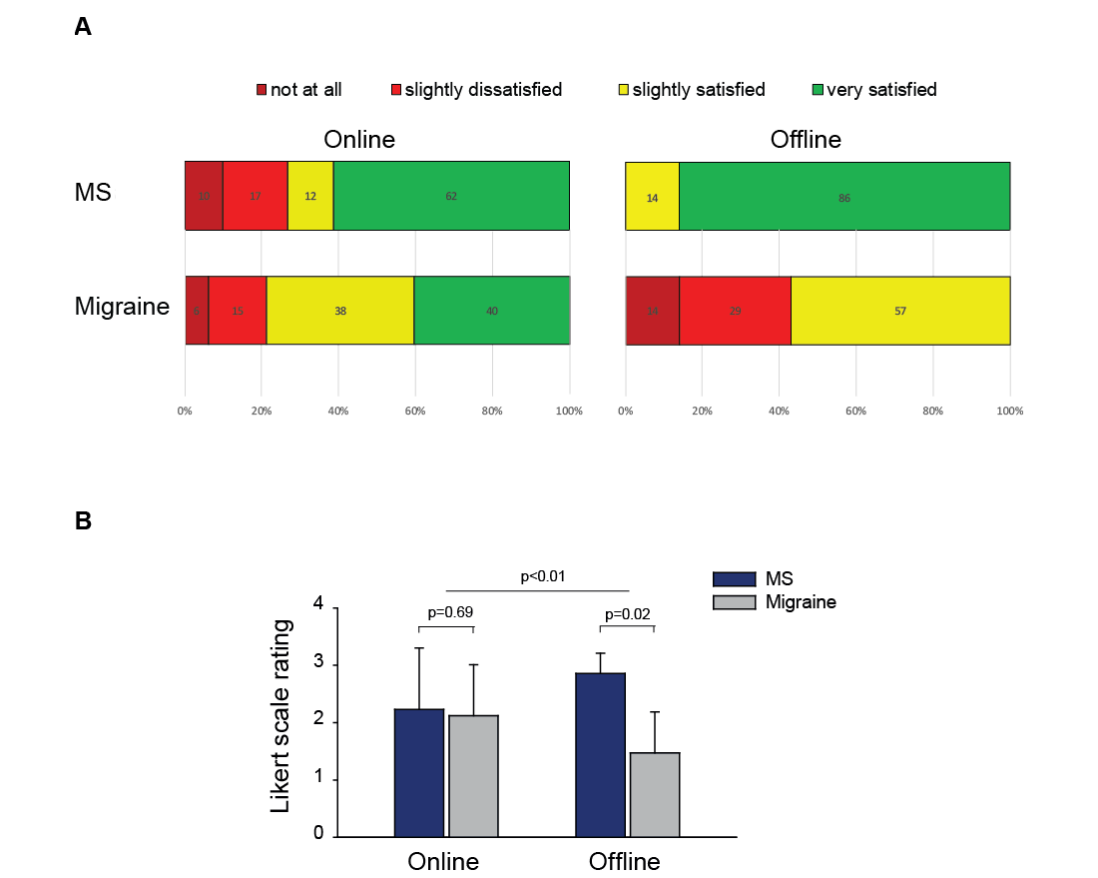
Neurologist satisfaction about information quality. A. Distribution of satisfaction ratings for retrieved information by information channel type and medical specialty (MS specialists, n=25, and migraine specialists, n=25). Physicians were asked to rate how satisfied they were about the retrieved information. Diagrams show percentage of information-seeking events for each score of the 4-point Likert scale used (from 0=not at all to 4=very satisfied); B. Differences in average satisfaction scores by information channel and medical specialty. *P* values refer to 2-way analysis of variance. MS: multiple sclerosis.

**Figure 5:**
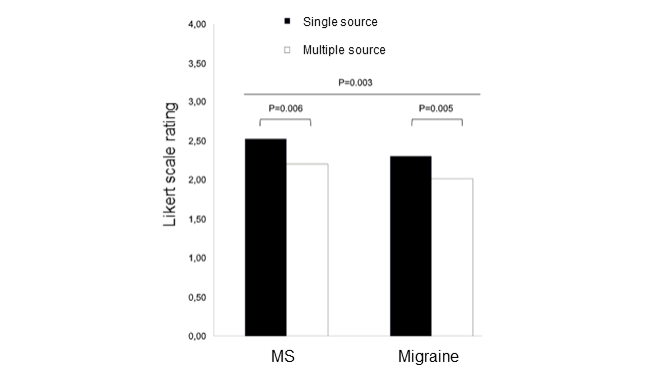
Neurologist satisfaction when using single or multiple sources. Average satisfaction ratings by medical specialty (MS specialists, n=25, and migraine specialists, n=25) and information sources adopted for each information-seeking event. *P* values refer to 2-way analysis of variance. MS: multiple sclerosis.

Physicians reported they were generally satisfied with the information retrieved (Figure 4A and 4B). Differences across channels and specialties in satisfaction ratings were similar to those observed for information retrieval ratings (Figure 4B, omnibus test, *F*=49.0; *P*=.01). In particular, migraine specialists were significantly less satisfied about offline channels compared with MS neurologists (*P*=.02). Conversely, satisfaction about information retrieved through online channels was similar in both specialty groups (*P*=.69). Finally, we observed reduced satisfaction when multiple sources were interrogated for a given information need (Figure 5, omnibus test: *F*=7.18, *P*=.001; single source vs multiple sources: *F*=12.02, *P*=.003). Such difference was observed among MS (*P*=.006) and migraine specialists (*P=*.005).

## Discussion

### Principal Findings

This research used an instant messaging phone app to investigate real-life needs, learning triggers, and information sources of Italian neurologists treating patients with either MS or migraine. This exploratory study provides a first time preliminary profiling of neurologists that could be useful for subsequent development of personalized approaches to medical education.

An interesting finding of this study is that seeking behavior is different between MS and migraine specialists, suggesting that the specific clinical scenario of specialization determines a distinctive pattern of interests, information needs, issues to be addressed, and preferred information sources. There are significant differences between current therapeutic management of MS and migraine, mainly due to the recent advances in the treatment strategies for MS. Our data on information needs and seeking behavior of neurologists seem to reflect the differences occurring in the therapeutic area in which physicians operate. Indeed, compared with MS specialists, migraine neurologists were less prone to engage in information-seeking behavior and, when they did look for medical information, they more frequently asked questions about therapy management rather than other topics. Furthermore, migraine physicians often needed to consult multiple information channels to find answers and more frequently relied on the use of public search engines rather than professional scientific repositories. Finally, satisfaction of migraine neurologists with offline channels (eg, including seminars, discussion with pharmaceutical sales representatives, congresses) was significantly lower than that of MS specialists.

A deeper analysis of resource use according to information needs indicates that neurologists have developed need- and time-specific research patterns that depend on the type and content of the piece of information searched for. Indeed, when starting new information seeking, physicians choose the most effective search process as well as the most suitable information source to answer that particular question. This requires neurologists to estimate the properties, reliability, weaknesses, and strength of each available channel. Moreover, the choice to use online or offline channels or a combination also depends on the environment in which the information need occurs (ie, during a patient encounter, at home, during a discussion with colleagues). For example, consistent with previous research [10,12,28], in our study group public search engines were exploited for initial exploratory researches or to rapidly meet simple information needs. This was particularly true when small pieces of information were needed to quickly answer simple questions raised in the course of medical interactions or educational events. In other words, it appears that these online contents are used when physicians need a prompt transfer of medical information into clinical practice. However, due to the difficulty in assessing the validity of the overwhelming amount of general public search engines or free online encyclopedia, physicians subsequently referred to more authoritative scientific resources, including PubMed or other indexed repositories of scientific literature and offline channels. On the other hand, to address complex issues needing articulated responses, a multichannel tree-like search process was generally adopted, in which both online and offline sources were used. The integrative use of online and offline channels often occurred when a single channel failed to meet a specific information need, as in the case of migraine specialists.

The complexity around information seeking and learning modalities recently gave rise to the principles of personalized education (PE). Like the emerging framework of PM [3,29,30], PE posits that the offer of educational services should reflect the specific content, timing, conciseness, ergonomics, and use channel needs at the point of use rather than the one-size-fits-all approach adopted so far [13,31,32]. In other words, PE involves identification of specific context-by-user information needs, optimization of educational activities, and design of user-centered learning [33,34]. In this context, medical information departments have the possibility to provide reliable medical content in a timely manner answering specific inquiries from specialists. However, in contrast with evidence from North American data [18], our findings show that the use of websites sponsored by pharmaceutical companies was marginal. On the other hand, recourse to pharmaceutical sales representatives was a prominent information source among offline channels, representing an important credibility asset for medical information services. In light of these data, customization of Medical Information services on the basis of specific needs, information gaps, and information-seeking behaviors for different therapeutic areas is both a challenge and an opportunity for pharmaceutical companies.

### Strengths and Limitations

This study was based on the use of the Physician Line app, a novel data collection tool that enables real-life observation and sharing of the information needs experienced by physicians. This unique feature allows granular, simultaneous data collection, thus minimizing recall bias. Therefore, the Physician Line app offers the opportunity to obtain results similar to those provided by ethnographic research, in which moderators interact with participants in their real-life environment. Moreover, this tool allows the identification of time- and context-specific information needs. Taken together, these observations indicate that the Physician Line app may represent a valuable strategy for identifying the educational needs and information search strategies of health care professional.

Some limitation of this research also needs to be acknowledged. First, the exploratory design involves a small sample size and a short observation period. Further, we lacked potentially important personal and contextual data that could bring further insight into information-seeking profiles among MS and migraine specialists.

### Conclusion

Our research shows that Italian neurologists practicing in different therapeutic areas experience different information needs, adopt different seeking behaviors, and refer to different information sources during their clinical practice and/or professional development. These findings suggest that an effective information delivery approach requires customization of both the information to be provided and the communication methods to be adopted, which must be tailored to the specific situations, needs, and requirements of health care professionals.

Based on this, identification of time- and context-specific needs as well as physician’ profiling appear to be essential steps to design personalized information delivery and medical education strategies for neurologists involved in different subspecialty therapeutic areas. This requires novel instruments enabling us to thoroughly assess physicians’ real-life information needs. This proof-of-concept study investigated the suitability of the Physician Line app as a tool to collect relevant data without affecting clinicians’ daily working routine and providing, at the same time, the opportunity for interaction between physicians and moderators. Based on these preliminary results, the Physician Line app appears to be a valuable tool enabling identification of specific context-by-user information needs, optimization of educational activities, and design of user-centered learning, in accordance with PE principles. Further research should be designed to evaluate the potential of this novel instrument for data collection in a larger sample population. Delivery of personalized, accurate, consistent, and timely information to physicians is essential to improve patient care.

## Appendix

Multimedia Appendix 1 Physician Line app items of the semistructured interview and Likert-based questions.

Multimedia Appendix 2 Information sources mentioned during the observation period.

Multimedia Appendix 3 Themes emerging from content analysis.

Multimedia Appendix 4 Use of information sources according to information needs.

## Abbreviations

MS: multiple sclerosis
PE: personalized education
PM: precision medicine
