# Supplementary material for: Information Needs and Information-Seeking Behavior of Italian Neurologists: Exploratory Mixed Methods Study
Source: J Med Internet Res. 2020 Apr 8;22(4):e14979. doi: 10.2196/14979 (PMC7177431; doi:10.2196/14979)
Supplement: Multimedia Appendix 1 [file jmir_v22i4e14979_app1.docx]

**Appendix 1.**

1. **Items of the Physician Line – App semi-structured interview**
2. Why did you initiate your search?
3. What content were you looking for?
4. Which source of information did you look at for this search?
5. Did you look at multiple information sources to get the content you were interested into?
   1. Yes |_| No |_|
6. If yes, please list all sources you looked at.
7. **Likert-scale questions administered with the Physician Line – App**

Have you found the content you were looking for?

1. Not at all 2. Partially Not 3. Neutral 4. Partially Yes 5. Yes

Are you satisfied by the information retrieved?

- - - 1. Not at all 2. Partially Unsatisfied 3. Slightly Satisfied 4. Very Satisfied
