# Supplementary material for: Information Needs and Information-Seeking Behavior of Italian Neurologists: Exploratory Mixed Methods Study
Source: J Med Internet Res. 2020 Apr 8;22(4):e14979. doi: 10.2196/14979 (PMC7177431; doi:10.2196/14979)
Supplement: Multimedia Appendix 2 [file jmir_v22i4e14979_app2.docx]

**Appendix 2. List of Information sources mentioned during the observation period**

| **Type** | **Sources** |
| --- | --- |
| Online | |
|  | Email newsletters |
|  | Video chat (ex. ground round with residents or external professionals) |
|  | Journal websites and general portals |
|  | Websites of scientific society |
|  | Medical and scientific journals |
|  | Online congresses |
|  | Other professional websites |
|  | Non-professional search engines (Google) |
|  | Portals specialized in scientific dissemination unsponsored by pharmaceutical companies |
|  | Indexed scientific repositories (Pubmed, Embase, Cochrane, etc.) |
|  | Websites sponsored by pharmaceutical companies |
|  | Institutional websites (hospital websites, government websites, etc.) |
|  | Physicians’ forum |
|  | Blogs focused on specific specialty area |
|  | Social networks (Facebook, Twitter, Sermo, etc.) |
|  | Instant messaging with colleagues (Whatsapp, Telegram, Messenger) |
|  | Web application supporting clinical practice (pharmaceutical codex, score) |
|  | Association of Patients (national, regional, local) |
|  | Public search engines (Google) |
|  | Patient forum |
|  | Portal of governmental and scientific institutions (ECTRIMS library) |
|  | Other (specify) |
| Offline | |
|  | Pharmaceutical sales representative |
|  | Physician management |
|  | Other company professionals (marketing) |
|  | Journals, original papers |
|  | Congresses |
|  | Round Table discussions/advisory Board |
|  | Promotional events |
|  | Practical training/stage/workshops |
|  | Hospital meetings |
|  | Educational events |
|  | Discussions with colleagues |
