# Supplementary material for: Information Needs and Information-Seeking Behavior of Italian Neurologists: Exploratory Mixed Methods Study
Source: J Med Internet Res. 2020 Apr 8;22(4):e14979. doi: 10.2196/14979 (PMC7177431; doi:10.2196/14979)
Supplement: Multimedia Appendix 3 [file jmir_v22i4e14979_app3.docx]

**Appendix 3. Themes emerged from content analysis.**

Data were collected by means of the Physician Line – App. Total queries are reported as N (%)*

| **Theme** | | **Item** | **Migraine Specialists**  **N=25** | **MS Specialist**  **N=25** | **p-value** |
| --- | --- | --- | --- | --- | --- |
| Clinical management | | | 21 (85.0) | 15 (60.0) | 0.053 |
|  | Posology/Treatment duration | |  |  |  |
|  | Drug safety and efficacy | |  |  |  |
|  | Medication comparison | |  |  |  |
|  | Drug mechanisms of action | |  |  |  |
|  | Treatment resistance/approaches to reduce resistance | |  |  |  |
|  | Guidelines | |  |  |  |
|  | Side effect management | |  |  |  |
|  | Use during pregnancy and breastfeeding | |  |  |  |
|  | Drug interaction/combination/synergy with other medications | |  |  |  |
|  | Contraindications and special considerations | |  |  |  |
|  | Storage and stability | |  |  |  |
|  | Other (specify) | |  |  |  |
| Drugs | | | 16 (65.0) | 17 (68.0) | 0.76 |
|  | Marketed drugs | |  |  |  |
|  | Drugs in the pipeline | |  |  |  |
|  | New drugs waiting for approval | |  |  |  |
|  | Other (specify) | |  |  |  |
| Diagnostic procedures | | | 10 (40.0) | 11 (44.0) | 0.77 |
|  | Identification of novel diagnostic methods/assays | |  |  |  |
|  | Diagnosis guidelines | |  |  |  |
|  | Information about local patient diagnosis processes | |  |  |  |
|  | Colleague consulting | |  |  |  |
|  | Other (specify) | |  |  |  |
| Congresses and educational opportunities | | | 7 (28.0) | 8 (32.0) | 0.77 |
| Disease epidemiology and physiopathology | | | 5 (20.0) | 8 (32.0) | 0.33 |
|  | Epidemiology | |  |  |  |
|  | Etiopathogenesis | |  |  |  |
|  | Novel discoveries about disease underlying mechanisms | |  |  |  |
|  | Other | |  |  |  |
| Patient-related topics | | | 2 (8.0) | 7 (28.0) | 0.06 |
|  | Physician-patient relationship | |  |  |  |
|  | Patient compliance/adherence to treatment | |  |  |  |
|  | Patient education about disease | |  |  |  |
|  | Patient education about therapy | |  |  |  |
|  | Information about resources and services supporting patient | |  |  |  |
|  | Patient assistance program | |  |  |  |
| Other | | | 1 (4.0) | 3 (12.0) | 0.61 |
| Pharmacoeconomics | | |  |  |  |
| Topical issues | | |  |  |  |
| Pharmaceutical companies and their activities | | | 0 (0.0) | 1 (4.0) | 0.99 |
|  | Information about companies committed to the specialty area | |  |  |  |
|  | Information about services provided by pharmaceutical companies | |  |  |  |
|  | Ongoing clinical trials related sponsored by a company | |  |  |  |
|  | Sponsored scientific activities (congresses, CME, courses) | |  |  |  |
|  | Sales representatives | |  |  |  |
|  | Other (specify) | |  |  |  |
