# Supplementary material for: Information Needs and Information-Seeking Behavior of Italian Neurologists: Exploratory Mixed Methods Study
Source: J Med Internet Res. 2020 Apr 8;22(4):e14979. doi: 10.2196/14979 (PMC7177431; doi:10.2196/14979)
Supplement: Multimedia Appendix 4 [file jmir_v22i4e14979_app4.docx]

**Appendix 4. Use of information sources according to information needs**

| **Information sources** | | **Information needs** | | | | | | | |
| --- | --- | --- | --- | --- | --- | --- | --- | --- | --- |
|  |  | **Total** | **Clinical management** | **Drugs** | **Diagnostic procedures** | **Disease epidemiology and physiopathology** | **Congresses** | **Patient-related topics** | **Pharmaceutical companies and their activities** |
|  |  | N=392 | N=169 | N=96 | N=34 | N=46 | N=23 | N=16 | N=3 |
| **ONLINE** | | **85%** | **87%** | **77%** | **82%** | **91%** | **91%** | **94%** | **67%** |
|  | Scientific literature repositories | 21% | 20% | 18% | 21% | 39% | - | 19% | 33% |
|  | General public search engines | 17% | 19% | 19% | 12% | 11% | 9% | 31% | - |
|  | Portals specialized in scientific dissemination | 9% | 16% | 5% | 9% | - | 4% | 6% | - |
|  | Websites of scientific society | 6% | 2% | 1% | 18% | 13% | 35% | - | - |
|  | Clinical and scientific journals | 6% | 6% | 6% | 12% | 7% | - | - | - |
|  | Institutional websites | 6% | 7% | 9% | 3% | 2% | - | - | - |
|  | Email newsletters | 5% | 4% | 6% | - | 9% | 13% | - | - |
|  | Web Application supporting clinical practice | 4% | 7% | 1% | 3% | - | - | - | - |
|  | Websites sponsored by pharmaceutical companies | 2% | 3% | 1% | - | - | 4% | 6% | 33% |
|  | Patients' association discussion forum | 1% | 1% | - | - | 2% | - | 13% | - |
| **OFFLINE** | | **15%** | **13%** | **23%** | **18%** | **9%** | **9%** | **6%** | **33%** |
|  | Pharmaceutical sales representatives | 5% | 4% | 10% |  | - | 9% | - | - |
|  | Consulting/discussion with colleagues | 3% | 3% | 1% | 9% | 2% | - | - | - |
|  | Congresses | 2% | 1% | 3% | 3% | 2% | - | - | - |
|  | Physician management | 1% | 1% | 1% | - | - | - | - | - |
|  | Other pharmaceutical professionals (marketing) | 1% | - | 2% | - | - | - | - | - |
